# System dynamics modeling in support of community-based decision-making to reduce opioid overdose fatalities

**DOI:** 10.3389/fpubh.2025.1616032

**Published:** 2025-07-28

**Authors:** Turner Canty, Matthew R. Lootens, Nasim S. Sabounchi, Rachel L. Thompson, Ayanava Ganguly, Nishita Dsouza, Steve Kilburn, Jill Beloch, Gary Hirsch, Terry T.-K. Huang, Nabila El-Bassel, David W. Lounsbury

**Affiliations:** 1 Center for Systems and Community Design and NYU-CUNY Prevention Research Center, Graduate School of Public Health and Health Policy, City University of New York, New York, NY, United States; 2 Princeton University, Princeton, NJ, United States; 3 School of Social Work, Columbia University, New York, NY, United States; 4 Chautauqua County Health Department, Mayville, NY, United States; 5 Broome County Mental Health Department, Binghamton, NY, United States; 6 Creator of Learning Environments, Wayland, MA, United States; 7 Department of Epidemiology and Population Health, Albert Einstein College of Medicine, Bronx, NY, United States

**Keywords:** systems thinking, system dynamics modeling, community-engaged research, implementation science, opioid use disorder

## Abstract

Both New York State (NYS) and the United States have experienced heightened levels of opioid overdose death and prevalence of opioid use in recent decades. While evidence-based practices (EBPs) to address opioid use and prevent overdose fatalities exist, their reach in many communities remains limited. Persistent systems-level barriers must be overcome to support and sustain effective EBP implementation. This paper describes the Systems Think Tank (STT), a community-engaged approach that promoted the use of systems thinking skills and system dynamics (SD) modeling for the purpose of local action planning and decision-making to select, employ, and monitor community-based strategies to prevent opioid overdose fatalities. A core modeling team launched the STT in support of the New York site of the HEALing Communities Study (NY HCS), a multi-site implementation research study funded by the HEAL Initiative. The modeling team worked collaboratively with purposively recruited NY HCS community coalitions located in counties across NYS. With the assistance of the modeling team, coalitions and their implementation teams explored SD modeling results and conducted strategy analyses using a web-based interface to simulate the local implementation of specific EBPs and inform action and sustainability planning. To describe the implementation of the STT, we reflect on our experiences with two NY HCS community coalitions and their implementation teams through two case studies. These case studies describe how SD modeling and systems thinking activities supported NY HCS coalitions during the CTH intervention by generating unique data and insights to inform coalition decision-making. We found that participation in the STT helped coalitions clarify the drivers of opioid overdose within their counties and identify potential effective strategies to mitigate overdose fatalities in the near future and long-term. The narratives presented in this paper may be useful for those incorporating SD modeling and systems thinking into community-engaged implementation research.

## Introduction

The ongoing opioid crisis has affected communities across the United States (US) (1–3) and has been exacerbated by the adulteration of the illicit drug supply by synthetic opioids (4, 5). Between 2018 and 2023, opioid overdose fatality rates increased 70 and 77% in the US and New York State (NYS), respectively (6). In response, many communities have sought to reduce opioid-related morbidity and mortality by adopting evidence-based practices (EBPs) to prevent fatal overdose and address opioid use disorder (OUD) (7, 8). However, increasing the reach of EBPs in community settings remains challenging due to limited availability of medications for opioid use disorder (MOUD) treatment, social stigma, lack of support systems, and other factors (9–12). Moreover, knowledge gaps remain around how to help communities build and sustain capacity to overcome these barriers (13, 14).

Establishing partnerships and coalitions with community members, decision-makers, and academic researchers is an effective way to direct knowledge and expertise into actionable, evidence-based strategies that address community-level barriers (15–17). The HEALing (Helping to End Addiction Long-term^SM^) Communities Study (HCS) was a large-scale implementation study to test the efficacy of the Communities That Heal (CTH) intervention, a collaborative, multi-phased action planning process aimed at reducing opioid overdose fatalities across affected communities in multiple states, including NYS (18, 19). The CTH intervention is a framework that assists communities in mobilizing the adoption and implementation of EBPs to reduce opioid overdose fatalities through a seven-phase community-led decision-making process (14, 20) in partnership with newly established or preexisting community coalitions based in a geographically diverse set of NYS counties (20).

NY HCS implemented the CTH intervention participating NY HCS coalitions convened a broad range of coalition members and local partners to help make decisions about which mix of EBPs would be most effective and sustainable in reducing opioid overdose fatalities in their community or county (9, 21). Coalitions also leveraged local resources and monitored available data on key indicators related to opioid overdose to inform this data-driven decision-making process (14). HCS leadership facilitated this collaborative process by supporting coalitions with funding, technical assistance, and access to resources to assist with implementation of the CTH intervention such as system dynamics (SD) modeling (14, 18).

System dynamics is a research methodology that allows for the analysis of complex systems to improve understanding of the endogenous feedback structures and nonlinear behavior within a system (22). SD modelers have used this approach to work with communities to address complex health problems by improving local understanding of the system, building consensus, and using models to simulate scenarios and test hypotheses (23, 24). SD modeling can help community partners identify, select, and coordinate implementation of solutions that account for a community’s available resources and capacities (25–27). The iterative nature of SD modeling can help communities build understanding and systems thinking skills over time to support data-driven decision-making through virtual experimentation to evaluate the implementation of EBPs such as those employed under NY HCS (28–30). In this paper, we define systems thinking skills broadly as the ability to understand how the components of a system interrelate, to identify the factors driving the system’s behavior, and to make reliable inferences about a system’s behavior by developing an understanding of the system’s structure (24).

Leveraging SD modeling and systems thinking practices, the Systems Think Tank (STT) was created as a component of NY HCS to help inform community coalitions’ data gathering, action and sustainability planning, and decision-making during the CTH intervention. The STT was engaged directly with NY HCS community coalitions during CTH phases 3 (Community Profiles and Data Dashboards), 4 (Community Action Planning), 5 (Implement and Monitor), and 6 (Sustainability Planning) (14). This community case study describes the results of the STT’s work with two NY HCS counties over a twenty-month period in 2022 and 2023.

## Methods

**Figure 1:**
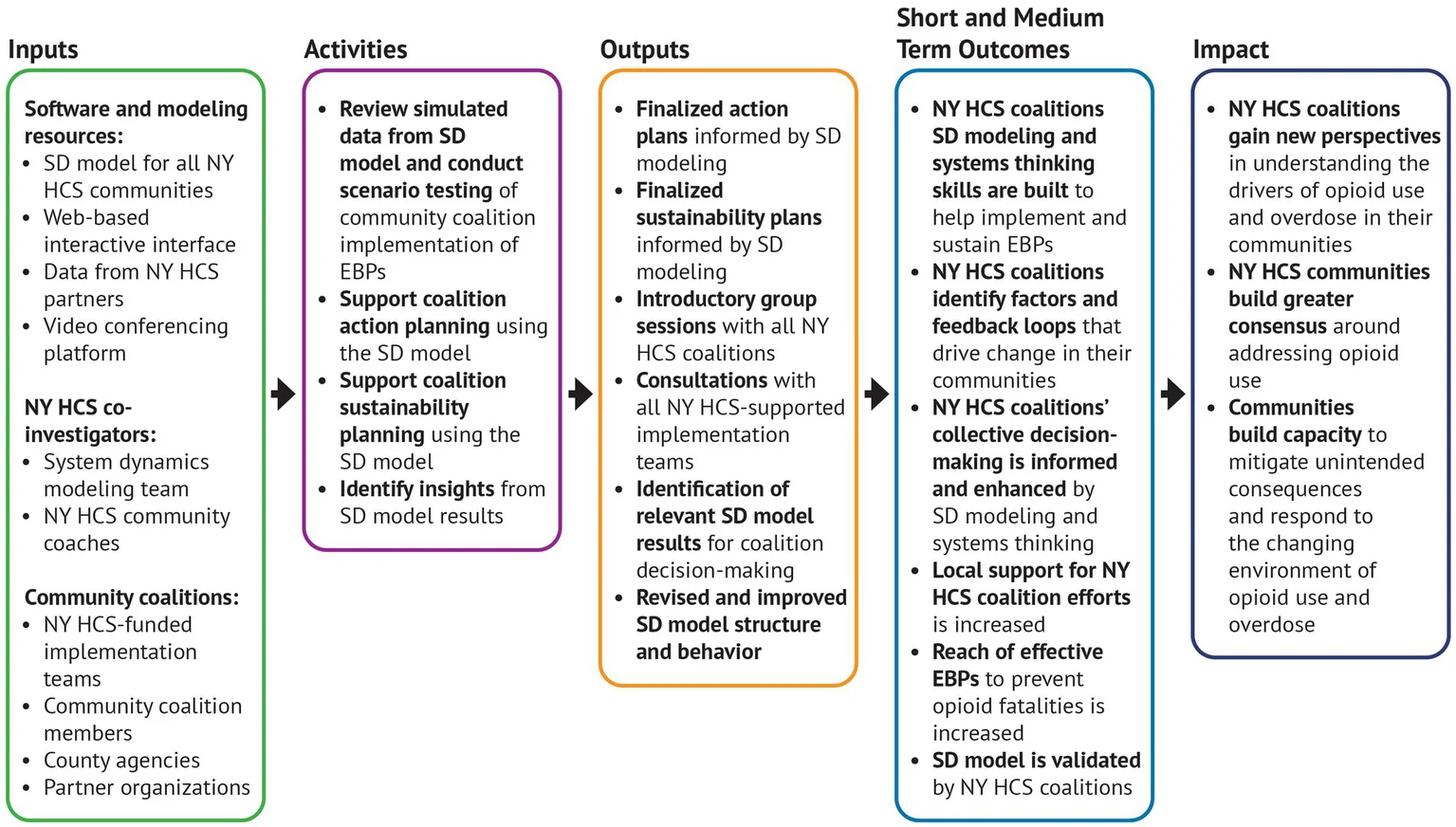
Logic model of Systems Think Tank implementation. This logic model presents the key components, activities, and outputs related to the implementation of the Systems Think Tank within the context of the New York HEALing Communities Study. EBP, Evidence-Based Practice; NY HCS, New York HEALing Communities Study; SD, System Dynamics.

The STT engaged NY HCS counties in a series of collaborative activities to foster systems thinking skills to inform the community-engaged intervention planning processes in the CTH intervention. A multidisciplinary and multi-institutional SD modeling team with expertise in SD modeling, qualitative methods, public health, community-engaged participatory research, and information science led these efforts. The modeling team worked with the NY HCS-supported implementation staff in each county (usually a Project Manager, Data Coordinator, and Community Facilitator) alongside members of the community coalitions and HCS-designated coaches who assisted the coalitions through the CTH intervention. See Figure 1 for an overview of the key activities, outcomes, and components of the STT.

At the center of the STT’s work was an SD model that captured the dynamics and drivers of opioid morbidity and mortality at the county and state level in NYS (see Supplemental material 1 for a diagram of the model’s structure) (31). The model’s structure was informed by prior published SD modeling of the US drug epidemic as well as by formative qualitative research conducted with NY HCS coalition members, who provided insights into the local challenges and opportunities in scaling evidence-based harm reduction and treatment practices (32–34). After calibration using over historical (2012–2021) opioid-related datasets from NYS, county, and federal agencies (e.g., annual overdose deaths, annual number of naloxone kits distributed), relevant literature, and expert opinion, the model simulated community-specific trends related to opioid overdose fatalities, OUD prevalence, local opioid supply and estimated potency, harm reduction and MOUD treatment capacities, among others (31).

**Figure 2:**
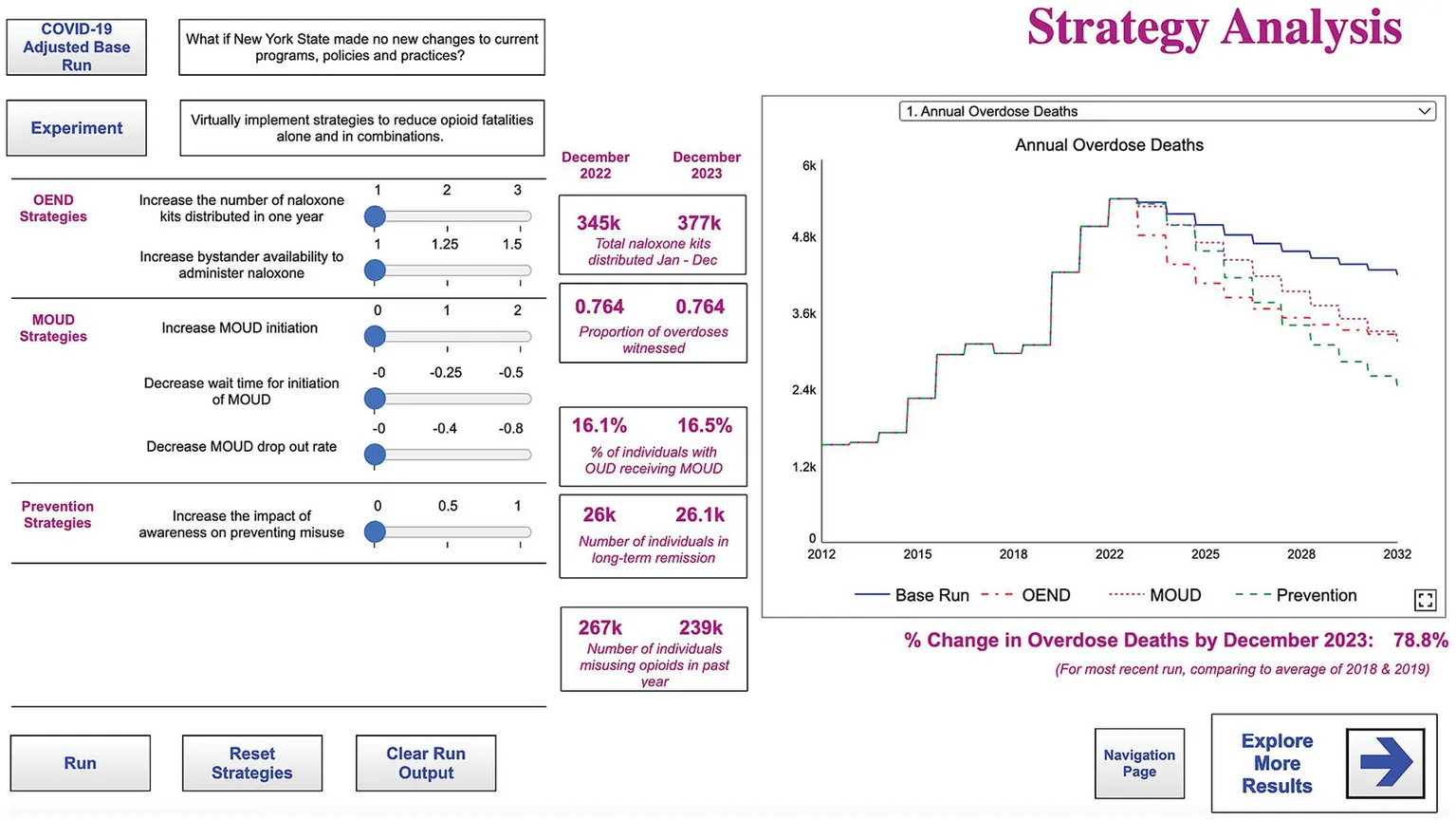
Strategy analysis page from the interactive interface. This interactive simulation tool was available through the interface for participants to conduct experiments to aid in planning and evaluation of evidence-based strategies to mitigate opioid-related outcomes in their communities. MOUD, Medication for Opioid Use Disorder; OEND, Overdose Education and Naloxone Distribution; OUD, Opioid Use Disorder.

The modeling team also developed an online portal to share information on the STT and give HCS coalitions the opportunity to access the SD model through a user-friendly interface to further develop systems thinking skills and insights for decision-making across the phases of the CTH intervention. This password-protected interface allowed coalition members to explore county-level outputs from the SD model and to compare potential short- and long-term trajectories for key variables. Users could conduct customized simulations by adjusting interface sliders that affected parameters in the underlying SD model and that corresponded to specific strategies (refer to the example page in Figure 2 for additional details). Strategies could be simulated alone or in combination from 2012 through 2032. HCS-supported EBPs guided which strategies were included in the interface (i.e., naloxone distribution, MOUD treatment availability, and treatment retention) (35). The interface was built with Stella Architect (36) and hosted on isee Exchange™ (37).

**Figure 3:**
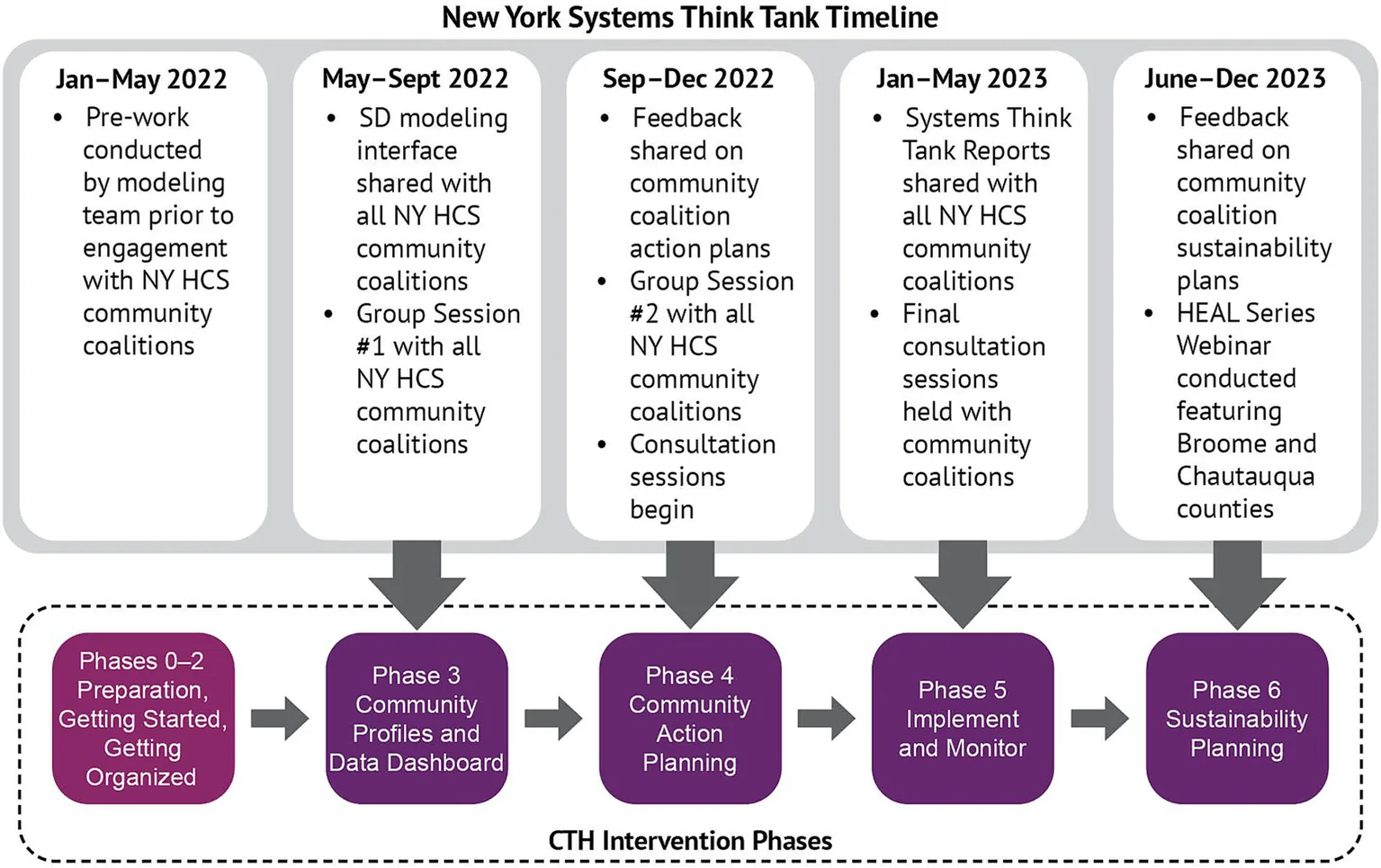
Overview of New York Systems Think Tank timeline. The implementation of the Systems Think Tank aligned with phases 3–6 of the communities that heal intervention. CTH, Communities That HEAL; HEAL, Helping to End Addiction Long-Term; NY HCS, New York HEALing Communities Study; SD, System Dynamics.

The STT began its work in CTH Phases 3 and 4 (Community Profiles and Data Dashboards and Community Action Planning) (Figure 3) (20). An initial goal was to introduce SD modeling and systems thinking to the NY HCS coalitions to encourage understanding opioid use as a systems-level problem and how EBPs might affect different parts of the system. To do so, we held two group workshops for key members of the community coalitions. The first workshop introduced the SD modeling team, showed the model’s ability to simulate historical data trends, and demonstrated how the model could help inform action planning and decision-making. The second workshop introduced the model interface and how it could be used to understand the drivers of opioid overdose and conduct strategy analysis. Supplemental material 2 provides the workshop agendas.

Toward the end of the action planning phase, we used model simulations to generate tailored reports describing the potential short- and long-term impacts of the EBPs that coalitions selected for implementation. These reports and the two group workshops established a foundation for engaging community coalition members and HCS implementation teams.

Throughout CTH Phases 4–6, HCS-supported implementation staff were able to request consultations with the SD modeling team. These consultations typically consisted of one-hour virtual sessions, allowing attendance by the geographically dispersed HCS implementation staff, modeling team, and coaches. The STT modeling team led these sessions and provided additional instruction in using the interactive portal, answered questions about the model’s underlying structure and the data used during its development, and facilitated discussion around community-specific drivers and causal factors described in the SD model. During initial consultations, the modeling team used the SD model to simulate coalitions’ action plans, explain revisions to the model, share data and model outputs related to specific coalition efforts. Follow-up consultations were held as requested and needed. The iterative nature of the consultations allowed the implementation teams to gain systems-level insights to share with their wider community coalitions to inform decision-making. The consultations also informed refinements of the SD model.

We collected data during all STT activities to capture coalitions’ experiences with the SD model and systems thinking insights. Group sessions and individual consultations were documented through recordings and structured notetaking forms completed by a dedicated notetaker. We also completed an ethnographic questionnaire after each consultation to record impressions, reflections, and observations using a rapid, pragmatic approach (38, 39).

The documented reflections and insights shared by the implementation teams from NY HCS community coalitions in Broome and Chautauqua Counties were used as core material for developing the case studies presented here. These coalitions also supported this paper by providing critical local data and clarifying and discussing their experiences engaging with the STT. These coalitions shared similar characteristics including number of partners and county demographics (Table 1) and provide comparable, yet contrasting, examples of incorporating systems thinking methodologies and SD modeling into community-level decision-making under a large-scale community-engaged implementation. The HCS-implementation teams for Broome and Chautauqua counties remained consistent for the length of their engagement with STT, and the program managers from these counties volunteered their time to conduct follow-up activities with the modeling team.

**Table 1:** Overview of coalition information and selected county demographics.

	Broome County	Chautauqua County
HCS coalition information		
HCS Urban/Rural Classification* (14)	Urban	Rural
Number of STT consultation sessions	6	4
Consultation frequency	39 Days	60 Days
Implementation team	Project Manager, Data Coordinator, Community Facilitator	Project Manager, Data Coordinator, Community Facilitator
Coalition partners (46)	30	30
Health-based	19 (63.4%)	17 (56.7%)
Criminal justice-based	4 (13.3%)	2 (6.7%)
Community-based	7 (23.3%)	10 (33.3%)
Other	0 (0.0%)	1 (3.3%)
Coalition established prior to HCS?	Yes (2016)	Yes (2018)
Demographics (2020)		
Population estimate (47)	198,683	127,657
White	158,674 (79.9%)	109,333 (85.6%)
Black or African American	12,684 (6.4%)	3,354 (2.6%)
American Indian or Alaska Native	556 (0.3%)	673 (0.5%)
Asian	9,372 (4.7%)	818 (0.6%)
Native Hawaiian and Other Pacific Islander	84 (<1%)	57 (<1%)
Other	3,975 (2.0%)	4,278 (3.4%)
Two or more races	13,338 (6.7%)	9,144 (7.2%)
Hispanic or Latino population (47)	10,285 (5.2%)	11,769 (9.2%)
Median age	40.0	42.9
Opioid-related morbidity and mortality (2022)		
Overdose deaths involving any opioid, age-adjusted rate per 100,000 population (41)	44.9	49.8
All emergency department visits involving any opioid overdose, age-adjusted rate per 100,000 population (41)	129.3	189.6

* The urban/rural designation was defined by the HCS study protocol based on county population density and geographic status (14). HCS, HEALing Communities Study; STT, Systems Think Tank.

## Results

### Case study 1: Broome County, NY

The Broome County HCS-supported implementation team developed systems thinking skills and SD modeling insights while engaging with STT during implementation of the CTH intervention. This implementation team was embedded in the larger Broome County coalition, which was supported by the Broome County Mental Health Department and other local partners. Six consultations sessions were held with the Broome County team, alongside email communication to ask questions or follow up on topics discussed during meetings. This sustained dialogue helped Broome County reinforce and further their understanding of systems thinking concepts and practices while building familiarity with the SD model.

#### Understanding the strengths, limitations, and complexities of SD modeling

During consultations, the implementation team members quickly grasped the SD modeling process, including running simulations and using the STT portal to evaluate potential effects of implementing EBPs. Broome County’s implementation team also showed a willingness to share insights from SD modeling with the broader coalition to help decision-making around community action planning and implementation. The Broome County team shared that opioid overdose and fatality rate trends generated by the SD model corroborated their observations of increased opioid overdose since the beginning of the COVID-19 pandemic. They questioned, however, the causes of this trend and indicated that the co-occurring arrival of fentanyl in the county may have been the primary driver of increased overdose fatality rates rather than social isolation and stay-at-home orders resulting from the COVID-19 pandemic. While the modeling team used data and available literature to estimate the effects of these orders, their role in driving model behavior was too extreme from the perspective of the Broome County implementation team. This view led to further discussion about changes observed in the potency of the illicit opioid supply in Broome County and how certain model structures could be adjusted to reflect these trends, with the goal of improving the validity of the SD model.

#### Addressing data needs

During the action planning phase of the CTH intervention, the Broome County team made frequent use of the interactive portal to conduct simulations, gather data to inform decision-making, and fill in gaps in available data related to opioid use and overdose. Specifically, they used the SD model to generate data estimates that would otherwise be unavailable in traditional surveillance data, such as future trends for OUD prevalence or exposure to illicit opioids. By leveraging these data to conduct scenario testing, the implementation team overcame data limitations to strategize about which EBPs would be most effective to implement in their county. The SD model’s ability to forecast future county-specific trends was of particular interest to the Broome County team, who was already aware of the need for a long-term perspective for estimating the effect of EBPs on opioid overdose fatalities. The implementation team was specifically interested in simulated data on community awareness of the dangers of opioid use, which they could use to help highlight the importance of outreach and awareness-building to the wider coalition. Unique data from the SD model was also generated through scenario testing, as the Broome County team generated and interpreted SD model outputs to identify complementary EBPs that would have the longest-term impact on opioid mortality in their county. For example, the implementation team found evidence that expanding access to MOUD while also expanding opioid overdose education and naloxone distribution (OEND) practices could work in tandem as part of the harm reduction strategy that would reduce fatal overdoses and give people using opioids more opportunities to seek treatment. During the sustainability planning phase of CTH, the Broome County implementation team also sought data to help them emphasize the need to sustain local support for effective EBPs over time, including data on overdose trends from the SD model that could indicate potential resurgences in overdose fatalities after a downturn.

#### Importance of maintaining community awareness

The Broome County implementation team emphasized that maintaining community awareness of the risks and harms associated with opioid use through communication campaigns and targeted outreach was central to their efforts. By “bringing attention to the voices of our impacted individuals” the implementation team sought to overcome barriers such as stigma to build capacity and potential local investment to sustain the coalition’s efforts after the conclusion of HCS (40). This was discussed frequently during consultations sessions, during which the modeling team described the community awareness structures in the model and guided the Broome County team to use the SD model interface to test different strategies to support awareness-building. As simulated in the SD model, increased community awareness can result in behavioral change of those using opioids by driving more people to seek treatment, cease, or otherwise reduce opioid use. The effect of awareness is also influenced by changes in opioid overdose mortality: an increase or decrease in fatal overdoses will cause a corresponding change to the effect of awareness in the model. Implementation team members saw maintaining awareness in the community as a key priority so that even when deaths diminished or fluctuated, support and capacity for their EBP strategies would remain steady. Using the SD model to visualize the effect of supporting community awareness in tandem with the implementation of other EBPs allowed the implementation team to share this finding with their wider coalition and informed their outreach efforts. During a consultation session, an implementation team member elaborated on this focus by sharing that awareness “must be built over time so that when deaths drop awareness is still maintained, maintaining awareness will be very important to keep momentum going.”

#### Reinforcing efforts in Broome County

SD modeling and systems thinking activities supplemented Broome County’s Phase 6 of the CTH (Sustainability Planning). As engagement with the STT progressed, the Broome County implementation team shared sustainability plans with the SD modeling team and used the SD model to generate new insights to reinforce their efforts over time. The implementation team members saw value in using these insights to help convene multiple partners to work toward shared goals, such as getting MOUD providers and harm reduction agencies to collaborate to improve treatment access. Modeling results were employed to support communications and outreach to bring community partners together by illustrating the potential impact of EBPs. For example, team members used the model’s interface during coalition meetings to illustrate how the impact of naloxone distribution on reducing opioid overdose fatalities could be improved if implemented in combination with other EBPs such as awareness-building. By visualizing these trends using the SD model and interface, the implementation team hoped to show the real-life benefits of implementing and sustaining multiple strategies over time. This was elaborated during a webinar featuring the Broome County program manager who shared that those interventions “have to work in tandem with one another and we would not have been able to highlight the visualization of that [to] give context and open up the dialogue without having access to the system dynamics model” (40).

Overall, the Broome County implementation team’s use of systems thinking in their own internal decision-making and external outreach to county partners aligned with the STT’s goal of supporting the CTH intervention. The implementation team made use of the SD model portal and the systems thinking approach to strategize and test the implementation of specific EBPs and build consensus on how to address the problem of opioid overdose fatalities. By sharing insights from the SD model, the Broome County team sought to gain wider support for coalition efforts to maximize impact over time on reducing opioid overdose fatalities.

### Case study 2: Chautauqua County, NY

The HCS-supported implementation team in Chautauqua County also participated extensively in the STT’s activities throughout the CTH intervention and used the SD model to investigate the underlying factors driving opioid overdose fatalities in their county. The Chautauqua County coalition and implementation team was supported by the Chautauqua County Department of Mental Hygiene and comprised a diverse set of members with training and expertise in addiction services, substance use treatment, community engagement, communications, and other skills. The Chautauqua County team attended four consultation sessions between November 2022 and May 2023 and afterwards continued to engage with the SD modeling team by email correspondence to discuss their experience using the SD model and its utility.

#### Understanding the strengths, limitations, and complexities of SD modeling

During their engagement with the STT, the Chautauqua County implementation team focused on exploring the SD model’s structure and sought to clarify the assumptions made by the modeling team about the dynamics of opioid use in their community. They also analyzed the data sources used to calibrate the model and investigated how simulations generated by the model compared with empirical data. They were initially skeptical about the accuracy of some simulated model data and aspects of the model’s structure. For example, they observed that simulating an increase in MOUD treatment initiation could lead to a short-term increase in overdose fatalities. This temporary increase in fatalities resulted from how the model captured the dynamic between opioid supply and demand: if some users in the population ceased or reduced use, the available supply per person using opioids would increase and lead to further fatal overdoses. In the view of the Chautauqua County team, this result was unrealistic as the county’s opioid supply was already saturated by the proliferation of fentanyl. In their view, while increased MOUD initiations could decrease demand for opioids, it was implausible that an increase in opioid availability due to the resulting leftover supply would lead to a substantial increase in overdose deaths. This was important feedback for the SD modeling team, and in subsequent versions of the SD model the effect of MOUD initiations on increasing available opioid supply was adjusted so that simulations would match these real-world observations.

While the Chautauqua County implementation team appreciated that the modelers attempted to capture the interacting dynamics of opioid supply, potency, and availability, they felt that some of these dynamics would be hard to forecast accurately due to insufficient local data. One implementation team member elaborated on this during a consultation session by sharing: “I do not think we should focus on the magnitude and the numbers because even the model cannot incorporate the details of that in real life, and on the street, things do not work this simply when it comes to opioid use and addiction.” The Chautauqua team also emphasized in later consultations that precise model projections were not as important as understanding the interdependent effects of specific feedback loops in the model and how those informed the overall model results.

#### Using the SD model to inform action planning, implementation, and monitoring

The Chautauqua County team also incorporated systems thinking insights to inform and support community action planning (CTH Phase 4). They used the interactive portal to conduct virtual experimentation and observed how some strategies, such as naloxone distribution, might have a limited effect on reducing overdose deaths if not combined with other strategies. For example, through model simulations, the Chautauqua team saw promising outcomes by combining naloxone distribution with increased MOUD prescribing. At the same time, the team anticipated challenges in increasing MOUD initiations, including the need for building public support. The SD model helped them recognize these interplaying dynamics and to set expectations for the effectiveness of EBP implementation and for realistic timelines.

During experimentation with the SD model, the Chautauqua County team noted that it could take years for some EBPs to have an impact on reducing opioid fatalities, while other EBPs would be more immediate. Since the SD model interface allowed a forecast horizon through 2032, they simulated several scenarios to see which combination of EBPs would have the most immediate and long-lasting impact. Through continued experimentation, the Chautauqua team continued to identify MOUD treatment initiation as a priority while also recognizing the importance of awareness-building strategies, a combination that could affect different parts of the system and have a wider impact on reducing fatality rates.

#### Overcoming challenges through an iterative process

After building an understanding of the unique contribution the SD model could provide towards informing their coalitions decision-making process, the Chautauqua team found utility in using the SD model to generate new insights and to explore the interplaying dynamics of factors related to opioid overdose in their county. While the implementation team noted that many of the findings generated by the SD model were sometimes difficult to parse, the team was appreciative of the ongoing conversations with the SD modelers and identified that the iterative process of updating the model with their input helped to generate further insights and ensure that the model remained relevant and responsive to their coalition’s efforts.

The Chautauqua County team also recognized the value of systems thinking and SD modeling when estimating the effects of different combinations of EBPs. While they were aware that the model’s simulated results were imperfect, they recognized the benefit of using the model to examine opioid overdose at the community-wide level and to refine their internal perspectives about how to address the problem. During engagement with STT, the Chautauqua County team used the model to try to explore how the trends around opioid use and overdose developed over time and understand why fatal opioid overdose continued to be prevalent in the community, despite past and present implementation of EBPs. One Chautauqua County implementation team member summed up his view by sharing that “the model really challenges us to think systemically in terms of the multiple interplaying dynamics and the feedback loops…it helps us define the complex reality of opioid use and addiction” (40). Overall, this sustained dialogue between the modeling team and the Chautauqua County coalition highlighted the importance of building understanding around SD modeling and systems thinking over time through an iterative process to ensure that SD models are relevant and responsive to the needs of communities.

## Discussion

The activities and outcomes of the STT described in these two cases suggest that systems thinking skills and tools can support data-driven decision-making within community coalitions and that this innovative approach could benefit other communities seeking to reduce opioid overdose fatalities. As a setting to support NY HCS, the STT enabled a systems thinking and SD modeling process with community coalitions to develop a shared understanding of the drivers of the opioid epidemic in relation to local conditions. Broome and Chautauqua Counties used STT resources, including the SD modeling interface, to examine the problem of opioid overdose from a systems-level perspective and develop key systems thinking skills, such as visualizing and interpreting trends in model outputs to identify insights about the casual relationships affecting opioid use their communities. We also found evidence that systems thinking and SD modeling uniquely informed decision-making about how best to improve reach of EBPs as part of the CTH intervention.

Both the Broome and Chautauqua teams used the SD model to identify key strategies that would prevent opioid overdose fatalities in their respective communities. However, while using the SD model to explore trends and test strategies, these two implementation teams assigned importance to different parts of the model’s structure. As modeling results are informed by the underlying conditions in the county and the historical data used to calibrate the model, the simulated impact of implementing specific EBPs could have a greater impact in one county compared to another. For example, increasing naloxone distribution might have a greater effect in a county where relatively little distribution had occurred historically. Notably, the Broome County team found the simulated effect of community awareness to be a key moderator of opioid overdose in their county. The implementation team concluded that maintaining awareness of the risks associated with opioid use was an important factor for preventing opioid overdose deaths over time. On the other hand, simulation results for Chautauqua County were less influenced by community awareness. Instead, the Chautauqua County implementation team focused their attention on the dynamics around opioid supply and potency in their county and associated the increasing trend of opioid fatalities observed in SD model with the proliferation of fentanyl into the local drug supply.

Continued dialogue with both teams also helped to identify aspects of SD modeling that were valuable for the coalitions and supported the iterative process of sharing information and insights. Through the activities of the STT, the SD modeling team elicited feedback to refine the model and make it more useful for coalitions’ efforts in EBPs implementation and prevention of overdose fatalities. This sustained dialogue fostered mutual understanding between the SD modeling team and implementation teams, ultimately resulting in an SD model that was more responsive to coalition and community needs.

The two counties used the SD model with differing goals and approaches and noted specific benefits and limitations in the modeling process. The Broome County team cited that using data from the SD model for outreach was beneficial in building public support and relationships in their county. This emphasis on outreach and finding synergies between partners’ efforts differed from the approach employed by the Chautauqua County team, who utilized the SD model more as an internal tool to investigate the dynamics of opioid overdose in their county. While the Chautauqua County team did question the precision of some modeling results, they saw that model simulations could help to illustrate the need to implement and sustain multiple EBPs to reverse reinforcing trends and increased opioid overdose fatalities seen in their county in the past decade (41). This helped Chautauqua to form hypotheses about why opioid overdose deaths remained locally high and test scenarios to identify the EBPs that would make the most significant long-term impact. Unlike the Broome County team, the Chautauqua team did not integrate these insights into outreach or coalition building efforts. Instead, they used this and other findings to help inform their internal understanding of the local drivers of OUD and overdose.

These experiences illustrate how SD modeling can be integrated into community decision-making using multiple approaches which emphasize different aspects of the model’s utility. The approach of the Broome County implementation team used modeling results to support their communications and outreach efforts in the community. In contrast, the Chautauqua County implementation team appreciated the SD model as a tool for internal deliberation but also found some aspects of the model to be inconsistent with their local perspective. Despite this, the Chautauqua implementation team still chose to engage with the SD modeling team to explain and address these concerns and help the modeling team refine the model to better reflect conditions in the county.

The STT demonstrated how SD modeling can be used to build mutual understanding and inform exploration of complex health problems, such as the opioid crisis, within diverse communities. This process required building trust on multiple levels, including trust in the model’s structure and behavior, the data used to calibrate the model, and the modelers themselves. Once trust in the systems thinking and SD modeling process was established, the implementation teams could test strategies and generate insights to inform their coalition’s decision-making and gain confidence in how best to leverage existing local capacities to effectively implement strategies with lasting impact.

Implementation scientists and substance use researchers have noted the need to better understand what explains long-term adoption of EBPs and how best to sustain these practices (13, 42). While SD modeling is not meant to be predictive, it can help build an understanding about how to achieve and sustain a desirable outcome while incorporating local and simulated data that might otherwise not be available. Future engagement with coalitions could build upon the modeling activities conducted during STT over a longer period while incorporating learnings from the coalitions in real-time to improve the model’s performance and provide relevant community-level data. Similar practices have been used to create collaborative learning environments, including in efforts to improve health systems (27, 43, 48).

## Limitations

Since engagement with the STT was voluntary, some NY HCS coalitions met with the STT less frequently, resulting in fewer opportunities to collect data and record insights from their experience of using the SD model and participation in systems thinking activities. Additionally, no data collection related to engagement with the STT or the SD model’s interface occurred outside of consultation sessions. As a result, the case studies presented in this paper were developed from available ethnographic and qualitative data and are not meant to be representative of every coalition’s experience of the STT, the online interface, or the utility of systems thinking for decision-making. The case studies described in this paper, as with other case studies, may not be generalizable to other populations or geographies and have not been evaluated for measures of data quality or comparability.

The COVID-19 pandemic restricted in-person access to NY HCS community coalitions and compromised original plans to conduct group modeling sessions and other in-person activities with the coalitions (44). Thus, ethnographic and qualitative data were collected during online engagement, using an adapted method informed by existing literature (9, 38, 45). As our method of data collection relied on input from the NY HCS-supported implementation staff, the experiences and perspectives of engaging with STT presented in the case studies may differ from those of the wider coalition membership. Lastly, no evaluation of fidelity, acceptability, or effect of the STT on NY HCS outcomes or the CTH intervention took place as this was outside of the NY HCS study design (14).

## Conclusion

The STT supported the NY HCS study by assisting communities in their local decision-making and planning to select and sustain EPBs for reducing opioid overdose fatalities. The technical assistance and support provided by the STT helped community coalitions investigate the drivers of opioid overdose from a systems perspective. By engaging in systems thinking and SD modeling activities, these coalitions were able to reflect on conditions in their county, conduct virtual experimentation with the SD model, and simulate the effects of various EBP strategies.

These two case studies show that creating a supportive learning environment to establish familiarity around SD and systems thinking methods is crucial towards establishing a relationship between the modelers and the community. The process of combining the expertise of the STT research team with the local perspectives of the NY HCS implementation teams and coalition members helped to foster a collaboration that provided a powerful decision-making tool to estimate the impact of EPBs at the community-level and built local capacity for systems thinking.

## Data Availability

The original contributions presented in the study are included in the article/Supplementary material. Further inquiries can be directed to the corresponding author.
