# Supplementary material for: System dynamics modeling in support of community-based decision-making to reduce opioid overdose fatalities
Source: Front Public Health. 2025 Jul 28;13:1616032. doi: 10.3389/fpubh.2025.1616032 (PMC12336117; doi:10.3389/fpubh.2025.1616032)
Supplement: Supplementary file 1 [file Data_Sheet_1.pdf]

## Session 1 Agenda:

Systems thinking basics: Embracing complexity, understanding feedback, leveraging capacity

June 9, 2022

| | |
|---|---|
| **10 mins** | Welcome and introductions |
| **20 mins** | Embracing Complexity: What's driving the opioid epidemic in our communities? |
| **20 mins** | Understanding Feedback: How are these drivers of the opioid epidemic inter-related (interdependent)? *Thinking in Loops!* |
| **20 mins** | Leveraging capacity: What is the most impactful way to scale evidence-based harm reduction, treatment, and safe prescribing practices in our communities? |
| **20 mins** | Small Group Discussion |
| **20 mins** | Small Group Report Back |
| **5 mins** | Future Think Tank Sessions and Strategy Consults |
| **5 mins** | Wrap Up and Session Evaluation |

## Session 2 Agenda:

Simulating high impact, sustainable 'reach' strategies in your community

October 6, 2022

| | |
|---|---|
| **10 mins** | Welcome and introductions |
| **45 mins** | Introducing the (new and improved) NY HCS Systems Dynamics Portal: What's driving opioid use, overdose risk, prevention, and treatment |
| **45 mins** | Fishbowl Exercise: How can simulation analyses support Phase 4 Action Planning to achieve and sustain desired EBP reach in your county (community)? |
| **15 mins** | Requesting Strategy Consults: How can we receive tailored simulation analyses to inform Phase 4 Action Planning for our county (community)? |
| **5 mins** | Wrap Up and Sessions Evaluation |
