# Supplementary material for: System dynamics modeling in support of community-based decision-making to reduce opioid overdose fatalities
Source: Front Public Health. 2025 Jul 28;13:1616032. doi: 10.3389/fpubh.2025.1616032 (PMC12336117; doi:10.3389/fpubh.2025.1616032)
Supplement: Supplementary file 2 [file Data_Sheet_2.docx]

**3. Key Terms and Abbreviations**

**Communities that Heal Intervention (CTH)**

A framework to assist communities in adopting evidence-based practices and conducting data-driven decision-making (1). The CTH intervention supports community coalitions to promote a common vision, shared goals, and actionable strategies. The HEALing Communities Study implemented the CTH intervention in both rural and urban communities to encourage adoption of EBPs and decrease the long-term effects of opioid use. CTH was adapted from the Communities that Care intervention which was implemented and evaluated in several states and has been seen as effective intervention to help coalitions select strategies to decrease substance use and other risky behaviors in their communities (2). CTH was delivered in 7 phases: (0) preparation, (1) Getting Started, (2) Getting Organized, (3) Community Profiles and Data Dashboards, (4) Community Action Planning, (5) Implement and Monitor, (6) Sustainability planning (1).

**Evidence-Based Practices (EBPs)**

Under HCS’ goal of implementing strategies that will reduce opioid-involved overdose fatalities, several practices with strong evidence in key domain areas were selected to be supported by the intervention. These domains were prevention, identification, treatment, and remission. Once the strategies were selected, a menu for HCS communities to select from was developed, known as Opioid-overdose Reduction Continuum of Care Approach (ORCCA). ORCCA sought to promote 3 primary strategies: overdose education and [naloxone](https://www.sciencedirect.com/topics/medicine-and-dentistry/naloxone) distribution (OEND), delivery of medication for opioid use disorder (MOUD), and prescription opioid safety (3).

**The HEALing Communities Study (HCS)**

A large-scale multisite study testing the Communities that Heal intervention over the course of five years (2019-2023) as facilitated by community coalitions working to address opioid overdose.^1^ HCS was implemented in 67 counties across 4 states: Ohio, Kentucky, New York and Massachusetts (4).

**Model Calibration**

A process to match a system dynamic model’s parameters and structure to observed and simulated structures and behaviors (e.g. outside data sources, observational research, etc.)(5).

**Model Validation:**

The formal process of testing and improving the structural and behavioral validity of the model. This iterative process is generally performed over the course of a model’s development and is validation often employs formal quantitative tools as well as qualitative evaluation. While similar to Model Calibration, validation also seeks to determine the model’s relevance to its target audiences and its overall quality in terms of structural validity (e.g., the model performs as its intended to perform) (6).

**System Dynamics (SD)**

An interdisciplinary method of examining complex systems through modeling and systems thinking (7). System dynamics can be used to gain insights and examine real-world problems and behaviors in complex systems that may present multiple barriers to traditional examination approaches and learning (8).

**Systems Thinking**

A term coined by Barry Richmond, meant to describe a paradigm and method for looking at the underlying structure of systems and making reliable inferences about system behavior (9). For the purposes of the Systems Think Tank, System Dynamics is a component of Systems Thinking, although debate has existed about where one begins and the other ends (10).

**References**:

1. Walsh SL, El-Bassel N, Jackson RD, Samet JH, Aggarwal M, Aldridge AP, et al. The HEALing (Helping to End Addiction Long-term SM) Communities Study: Protocol for a cluster randomized trial at the community level to reduce opioid overdose deaths through implementation of an integrated set of evidence-based practices. Drug and Alcohol Dependence. 2020 Dec;217:108335.

2. Oesterle S, Kuklinski MR, Hawkins JD, Skinner ML, Guttmannova K, Rhew IC. Long-term effects of the communities that care trial on substance use, antisocial behavior, and violence through age 21 years. Am J Public Health. 2018 May;108(5):659–65.

3. Winhusen T, Walley A, Fanucchi LC, Hunt T, Lyons M, Lofwall M, et al. The Opioid-overdose Reduction Continuum of Care Approach (ORCCA): evidence-based practices in the HEALing Communities Study. Drug and Alcohol Dependence. 2020 Dec;217:108325.

4. Sprague Martinez L, Rapkin BD, Young A, Freisthler B, Glasgow L, Hunt T, et al. Community engagement to implement evidence-based practices in the HEALing communities study. Drug and Alcohol Dependence. 2020 Dec;217:108326.

5. Oliva R. Model calibration as a testing strategy for system dynamics models. European Journal of Operational Research. 2003 Dec;151(3):552–68.

6. Barlas Y. Formal aspects of model validity and validation in system dynamics. System Dynamics Review: The Journal of the System Dynamics Society. 1996;12(3):183–210.

7. Sterman JD. System dynamics modeling: tools for learning in a complex world. California Management Review. 2001 Jul;43(4):8–25.

8. Sterman JD. Business dynamics: systems thinking and modeling for a complex world. Nachdr. Boston: Irwin/McGraw-Hill; 2009. 982 p.

9. Richmond B. Systems thinking/system dynamics: Let’s just get on with it. System Dynamics Review. 1994 Jun;10(2–3):135–57.

10. Amissah M, Gannon T, Monat J. What is Systems Thinking? Expert Perspectives from the WPI Systems Thinking Colloquium of 2 October 2019. Systems. 2020 Feb 27;8(1):6.
