# Supplementary material for: System dynamics modeling in support of community-based decision-making to reduce opioid overdose fatalities
Source: Front Public Health. 2025 Jul 28;13:1616032. doi: 10.3389/fpubh.2025.1616032 (PMC12336117; doi:10.3389/fpubh.2025.1616032)
Supplement: Supplementary file 3 [file Image_1.pdf]

Structure of the System Dynamics Computational Model

The core stock-and-flow structure and the corresponding feedback loops of the computational model developed for New York HCS counties to simulate the dynamics of the opioid epidemic are displayed in diagrams D1 through D6 on the following pages.

The behavior of the computational model is defined by a structure of stocks and flows driven by feedback loops. The stocks, depicted by boxes, represent the accumulation of a variable at a specific point in time (e.g., individuals with opioid use disorder not in treatment). The flows, shown as pipes with valves, signify the rate of change of the corresponding stock variable (e.g., initiation of sporadic opioid use). Links connect different stocks and variables as shown in the model diagram create feedback within the system, resulting in feedback loops. Feedback loops are shown with labels R for reinforcing and B for balancing loops. Polarity (+/-) signs denote the nature of the causal relationship between variables: positive, meaning they move in the same direction, or negative, indicating they move in the opposite direction. Reinforcing feedback loops cause exponential growth (or reduction) and accumulation within the system, while balancing loops help to stabilize the system by bringing variables into equilibrium. To further explore the structure and development of this model please review Sabounchi et al. 2025 (1) or visit the following webpage:

exchange.iseesystems.com/public/cscd/ny-hcs-system-dynamics-model-interactive-portal/index.html#page1

1. Sabounchi NS, Thompson RL, Lootens MR, Lounsbury DW, Hirsch G, Blevins D, et al. System dynamics modeling to inform implementation of evidence-based prevention of opioid overdose and fatality: a state-level model from the New York HEALing Communities study. International Journal of Drug Policy. 2025 Aug;142:104843. doi:10.1016/j.drugpo.2025.104843

D1: Individuals Using Opioids & Overdose Deaths

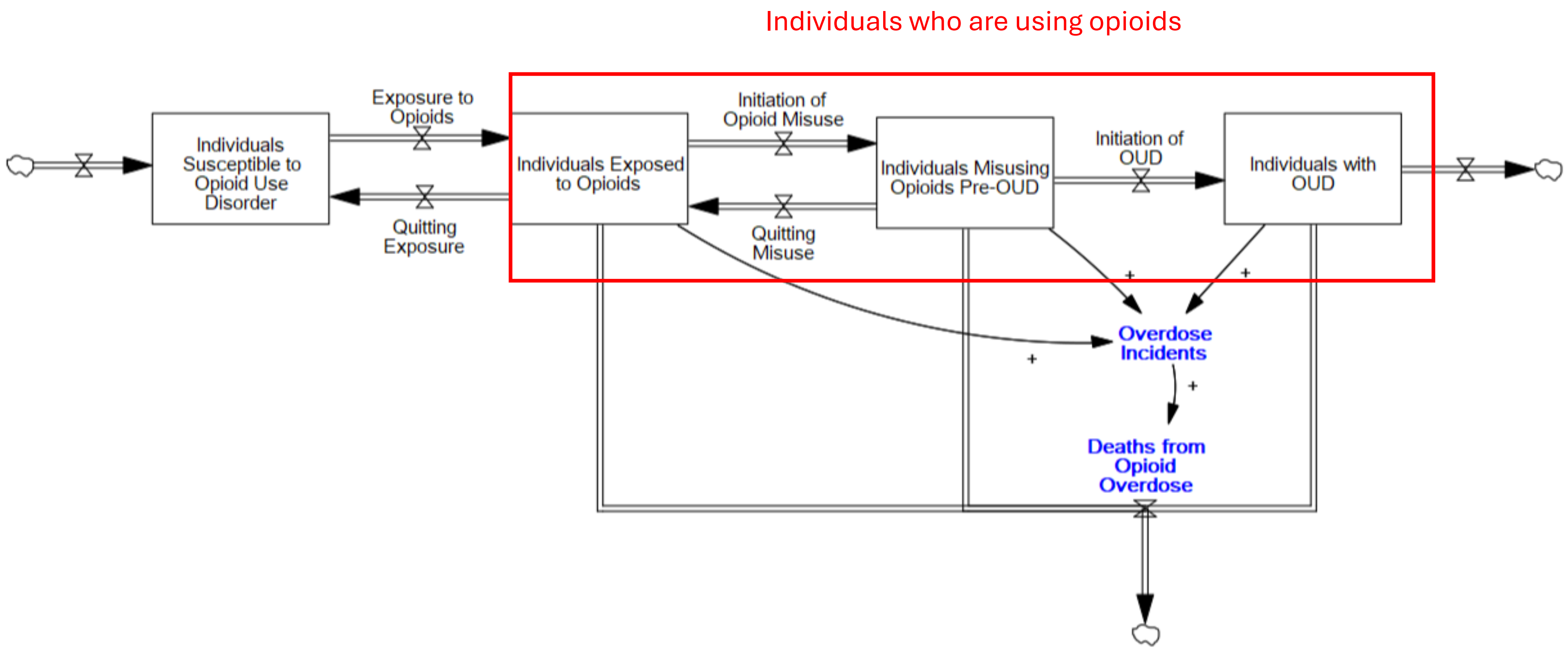

D2: Social Influence Loop

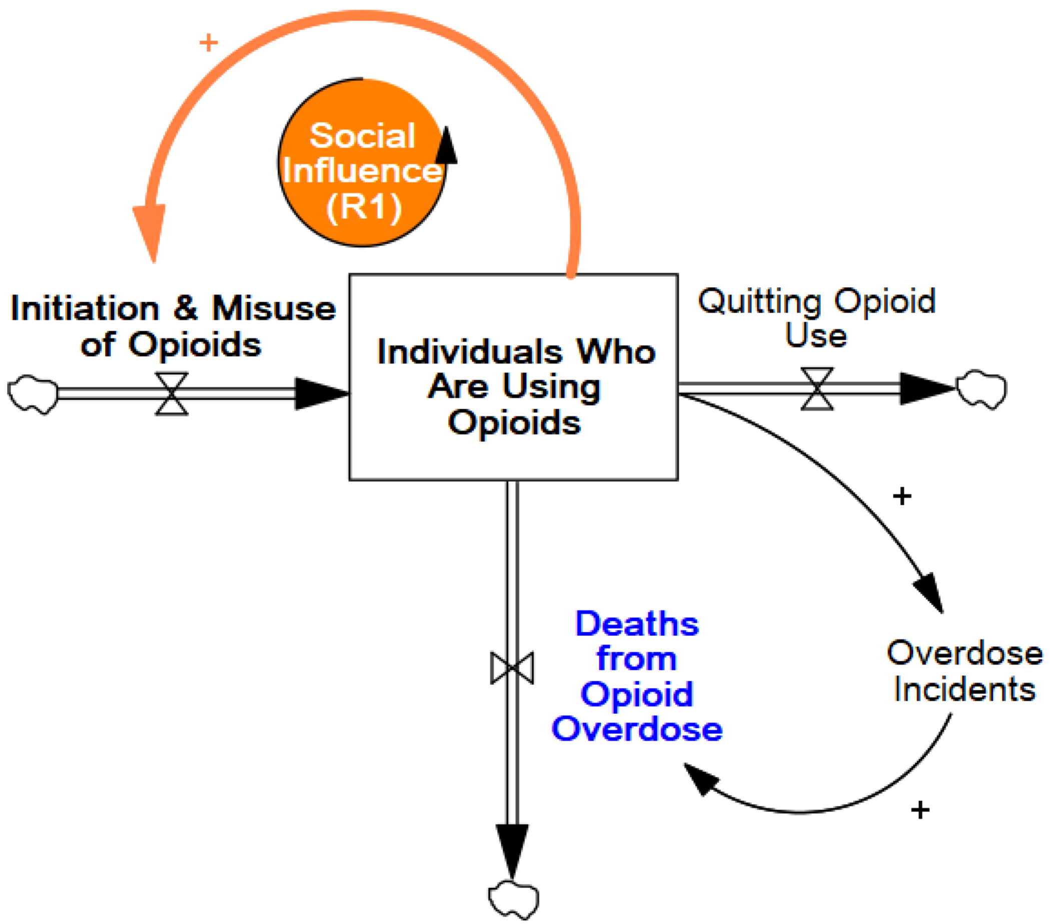

D3: Supply and Demand & Opioid Use

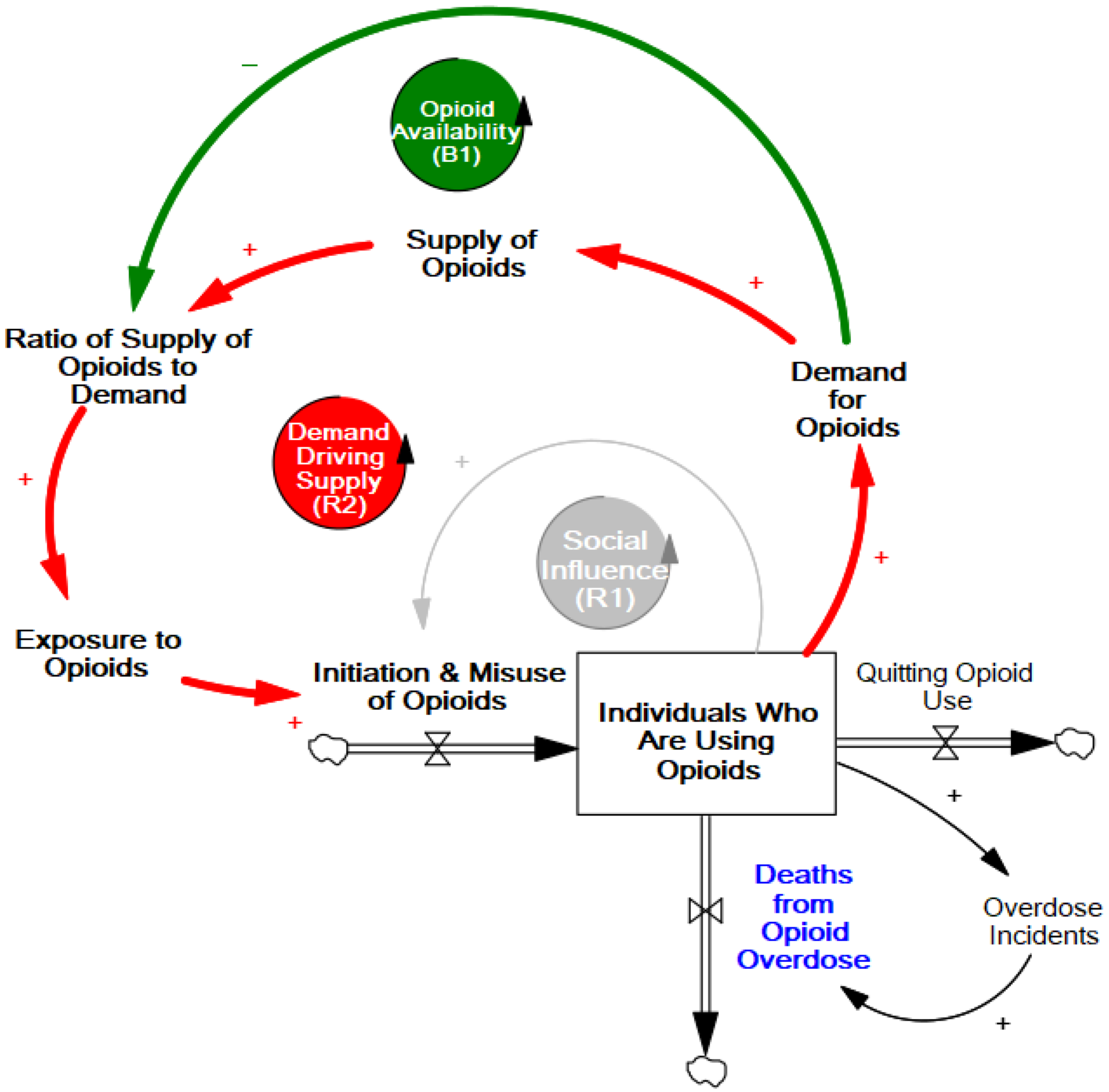

D4: Community Awareness & Opioid Use

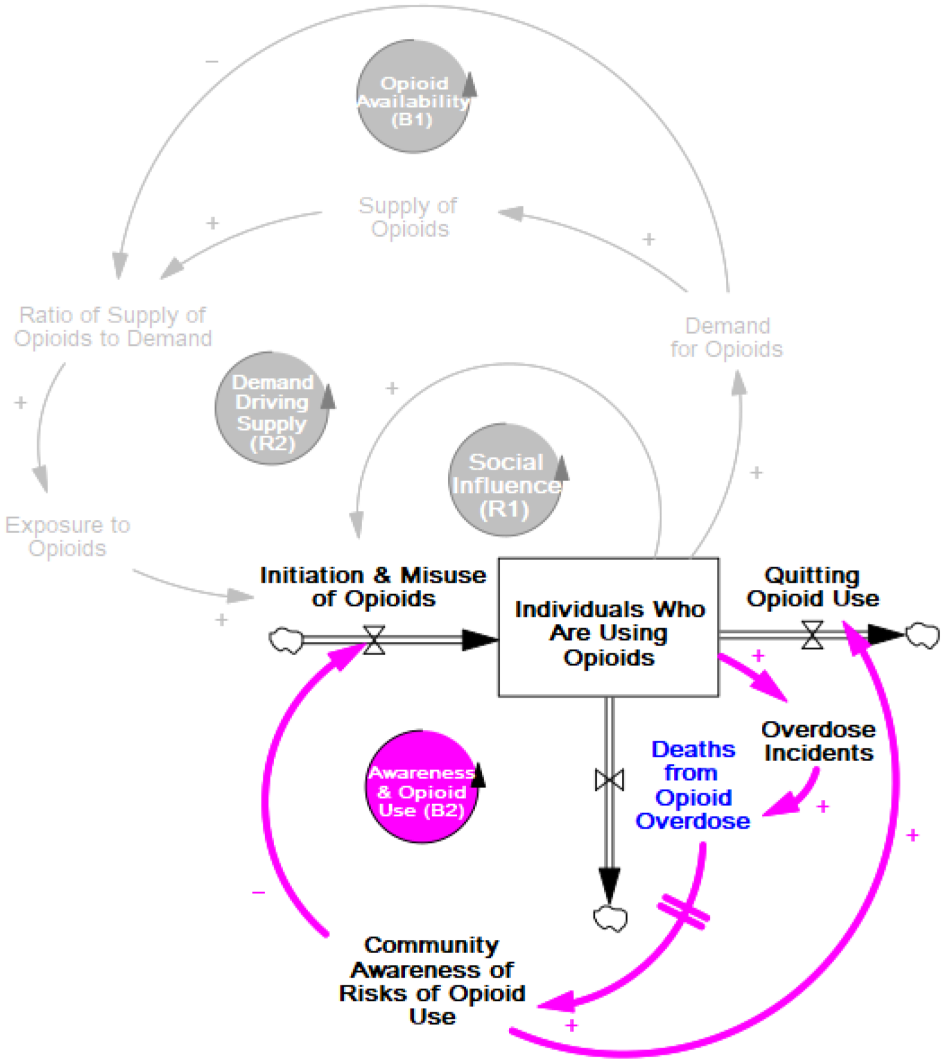

D5: Supply, Demand, and Overdose Risk

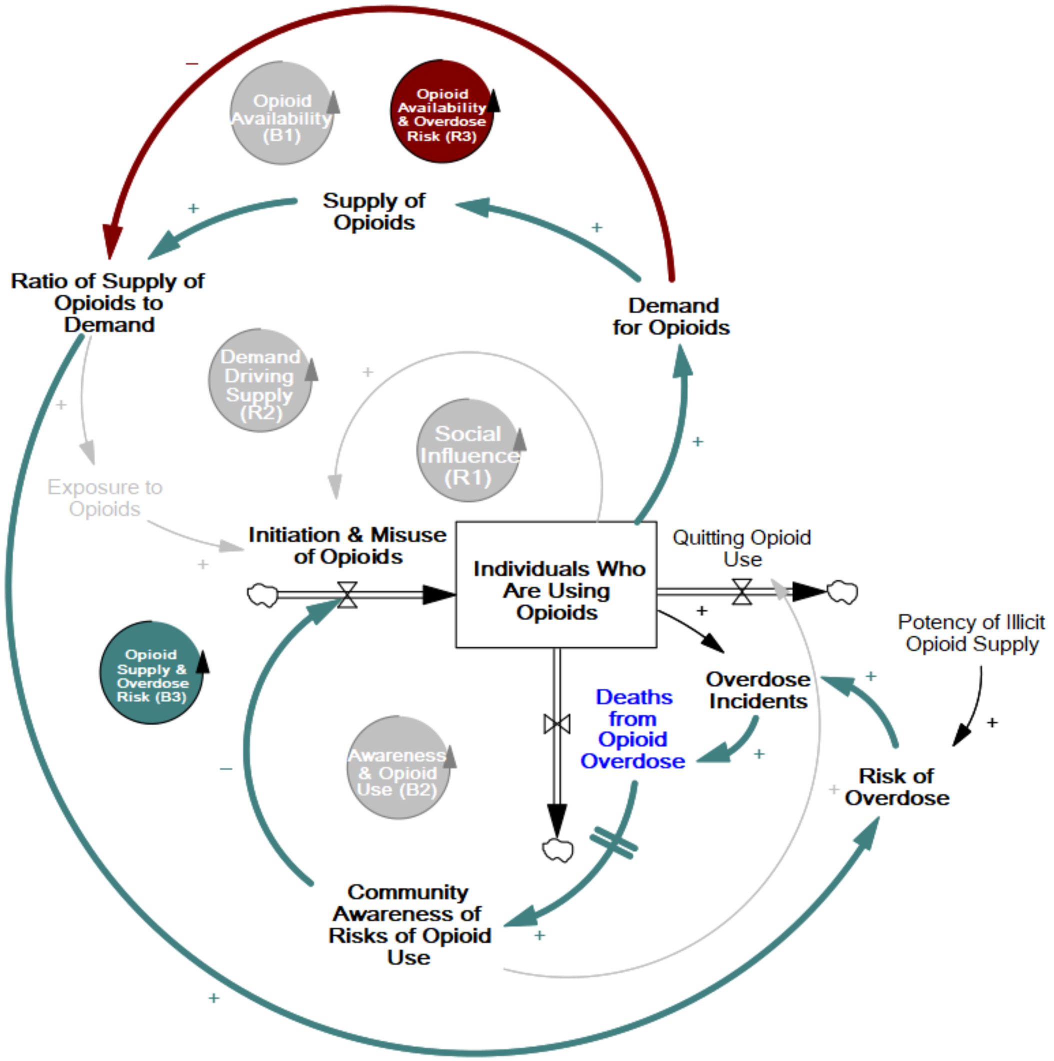

D6: Overall Structure of the System Dynamics Computational Model*

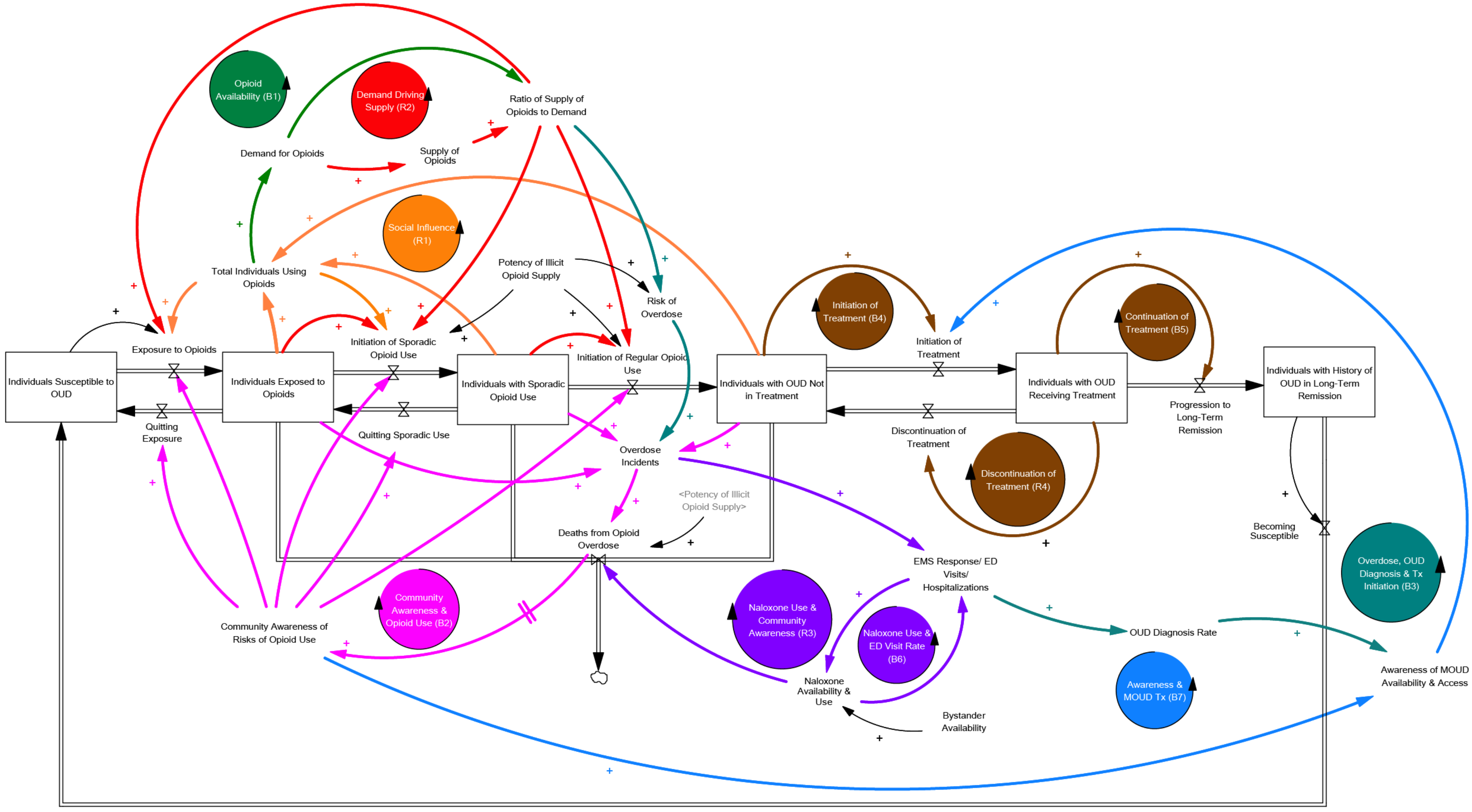

*Adapted from Sabounchi et al, 2025 (1). Reproduced with permission from the authors.

1. Sabounchi NS, Thompson RL, Lootens MR, Lounsbury DW, Hirsch G, Blevins D, et al. System dynamics modeling to inform implementation of evidence-based prevention of opioid overdose and fatality: a state-level model from the New York HEALing Communities study. International Journal of Drug Policy. 2025 Aug;142:104843. doi:10.1016/j.drugpo.2025.104843
